# On the dynamics of cortical development: synchrony and synaptic self-organization

**DOI:** 10.3389/fncom.2013.00004

**Published:** 2013-02-15

**Authors:** James Joseph Wright, Paul David Bourke

**Affiliations:** ^1^Department of Psychological Medicine, Faculty of Medicine, The University of AucklandAuckland, New Zealand; ^2^Liggins Institute, The University of AucklandAuckland, New Zealand; ^3^iVEC@UWA, University of Western AustraliaPerth, WA, Australia

**Keywords:** synchronous oscillation, cortical development, synaptic organization, cortical response properties, cortical information flow

## Abstract

We describe a model for cortical development that resolves long-standing difficulties of earlier models. It is proposed that, during embryonic development, synchronous firing of neurons and their competition for limited metabolic resources leads to selection of an array of neurons with ultra-small-world characteristics. Consequently, in the visual cortex, macrocolumns linked by superficial patchy connections emerge in anatomically realistic patterns, with an ante-natal arrangement which projects signals from the surrounding cortex onto each macrocolumn in a form analogous to the projection of a Euclidean plane onto a Möbius strip. This configuration reproduces typical cortical response maps, and simulations of signal flow explain cortical responses to moving lines as functions of stimulus velocity, length, and orientation. With the introduction of direct visual inputs, under the operation of Hebbian learning, development of mature selective response “tuning” to stimuli of given orientation, spatial frequency, and temporal frequency would then take place, overwriting the earlier ante-natal configuration. The model is provisionally extended to hierarchical interactions of the visual cortex with higher centers, and a general principle for cortical processing of spatio-temporal images is sketched.

## Introduction

During its embryological development the mammalian brain differentiates from a group of stem cells into an organized form ready to begin a life-long adaptive interaction with signals from the sensory environment. At the beginning of extra-uterine life, despite exposure to a limited milieu, it is somehow already organized to begin this engagement, as tho a matrix of connections has formed in which signal flows are pre-adapted to learn specific recurring patterns of the experiential world. A large body of work, following the pioneering work of Hubel and Wiesel (1959), has addressed just this issue, taking as the main target for research the primary visual cortex (V1). The majority of this work has sought to understand the emerging connections in terms of stimulus “features”—that is, elementary properties of sensory stimuli—rather than as a process independent of sensation until the post-natal stage. Our approach depends on alternative assumptions. Here we summarize and extend our earlier work (Wright et al., 2006; Wright and Bourke, 2008, 2013; Wright, 2009, 2010) relating the basic dynamics of neuron firing and competition among developing neurons for the resources needed for their growth, to the emergent connections at birth.

Our model draws on two recent experimental observations. Firstly, neurons in neonatal cerebral cortical slices show increased apoptosis when their capacity to enter into synchronous firing is disrupted by pharmacological means (Heck et al., 2008). Secondly, embryonic neurons developing *in vitro* develop synchronous firing, and as their growth proceeds, show self-organization into “small world” networks (Downes et al., 2012). We propose that the synchronous firing and protection from apoptosis are directly causally related, because during cortical embryogenesis there is competition among developing neurons and synapses, which, although mediated by trophic factors (Harris et al., 1997; van Ooyen and Willshaw, 1999; van Ooyen, 2001) is ultimately a competition for available metabolic energy and/or some other scarce resource needed to promote metabolism (Montague, 1996; Thomaidou et al., 1997). We suppose that pre-synaptic pulse synchrony increases uptake of critical metabolic resources by some action not presently specified, and we argue that the assembly of cells that maximizes synchronous firing, and thus energy uptake, is also that which has the minimum metabolic cost per neuron in the length of axonal connections—the combination optimum for their survival.

Synchronous oscillation of pulses and local field potentials is a ubiquitous aspect of cortical activity (Eckhorn et al., 1988, 1990; Gray et al., 1989; Bressler et al., 1993; Singer, 1999) and has been proposed as a mechanism solving the “binding problem” of perceptual grouping and cognitive processing (Eckhorn et al., 1990; Singer, 1999; Crick and Koch, 2003). “Synchrony” refers to the broadband cross-correlation of neuron firing and field potentials at zero time-lag. The mechanism of origin of synchrony itself is controversial. In this paper we rely on an explanation that appears best applicable to the synchrony seen in neuron cultures, brain slices, or the early embryonic brain, and depends on a universal property of networks with summing junctions, including dendrites (Robinson et al., 1998; Wright et al., 2000; Chapman et al., 2002). This type of synchrony appears in simulations that also accurately reproduce spectra, cross-correlations, and excitatory/inhibitory timings characteristic of activated cortex (Wright, 2009, 2010).

### Geometry of response organization in the developed brain

Since the discovery that individual cells in the primary visual cortex (V1) respond with an orientation preference (OP) to visual lines of differing orientation (Hubel and Wiesel, 1959), analysis of the response organization and its relationship to cortical function has remained both conceptually influential and controversial (von der Malsburg, 1973; Willshaw and von der Malsburg, 1976; Swindale, 1996). The surface organization of OP in V1 has recently been compared with appropriate random surrogates, and shown to exhibit significant hexagonal rotational periodicity, in which each roughly delineated macrocolumnar unit exhibits all values of OP arrayed around a pinwheel (Paik and Ringach, 2011; Muir et al., 2011). Varying chirality and orientation of the pinwheels achieves continuity of OP at the columnar margins, thus producing zones of irregular but continuously varying OP, known as linear zones and saddles.

Some species exhibit little or no sign of this hexagonal and continuous ordering, and because of the marked interspecies variation, serious doubt has been expressed that the pattern is of functional significance at all, since species showing little such organization have no apparent deficit in vision (Horton and Adams, 2005). Interspecies variation seems, in part, to be related to both variation in size of V1 between species, and a relative constancy of the size of macrocolumns, independently of species. Measurements of the average distance of separation of OP singularities (the singularity taken as demarcating the center of a macrocolumn) show this distance to be relatively constant over a 40-fold variation of body size, and related size of V1 (Kaschube et al., 2010; Keil et al., 2012). Models using symmetry arguments indicate that macrocolumns must undergo divisions during cortical development to maintain uniform surface density of singularities (Wolf and Geisel, 1998; Oster and Bressloff, 2006). Kaschube and colleagues conclude that self-organization has canalized the evolution of the underlying OP maps into a single common design—subject to the proviso that, from further symmetry arguments, this can only be the case where long-range interactions between developing macrocolumns, suppressing some possible connections, can take place. Thus, in animals with very small V1, this organization breaks down, creating a “pepper and salt” OP map pattern (Meng et al., 2012).

### The superficial patch system

A related puzzle of V1 organization is posed by the superficial patch system. This system, composed of relatively long-range, largely excitatory (Hirsch and Gilbert, 1991; McGuire et al., 1991) patchy connections (Gilbert and Wiesel, 1979; Rockland and Lund, 1983) is ubiquitous in cortex (Muir and Douglas, 2011) and has a functional relationship to OP. Patchy connections develop before sensory afferents reach the cortex (Price, 1986; Callaway and Katz, 1990; Durack and Katz, 1996; Ruthazer and Stryker, 1996) but do not arise or terminate in the vicinity of OP singularities. Instead, near singularities, connections are apparently diffuse and local (Sharma et al., 1995; Yousef et al., 2001; Mariño et al., 2005; Buzás et al., 2006; Muir and Douglas, 2011). Patchy connections link areas of common OP (“like-to-like”) over distances several times the diameter of a macrocolumn (Gilbert and Wiesel, 1989; Buzás et al., 2006; Muir et al., 2011), are periodic on roughly the same interval as OP, and are largely patch-reciprocal (Rockland and Lund, 1983; Angelucci et al., 2002). It has been shown that development of patchy connections must depend on the supply of organizing information from the neural field, and is not explicable from considerations of local neural growth *per se* (Muir and Douglas, 2011). Just as for maps of response properties, there is variation of patchy connection orderliness between species. Muir et al. (2011) have pointed out that those species with less orderliness have smaller visual cortices and/or less defined organization of “like-to-like” connections—an argument congruent with the findings on brain size, orderliness of response maps, and surface density of OP singularities cited above (viz. Kaschube et al., 2010; Keil et al., 2012, etc.).

### Problems of standard models of feature responses

Explanation of organization of OP has been undertaken in a group of now-classical theories, which we will refer to as “standard models,” following the comparative description of Swindale (Swindale, 1996). Descriptive dimension reduction methods (Kohonen, 1982; Durbin and Willshaw, 1987; Durbin and Mitchison, 1990) show that the response maps of OP, eye preference (OC), direction preference (DP), and spatial frequency preference (SF) are consequences of requiring continuity and completeness of representation of each response property, in a two-dimensional representation in which every type of response property occurs within any small area on the surface of V1 (Swindale, 1996; Carriera-Perpiñán et al., 2005). The same ordering is also explained as a consequence of competitive Hebbian learning among small neighborhood assemblies of excitatory neurons, driven by spatially filtered cortical noise. Separate spatial filters each distinguish a type of response, and total synaptic gain is conserved during the training (Grossberg and Olson, 1994).

Classical standard models depend on seeding with oriented lines, in one way or another (von der Malsburg, 1973; Swindale, 1982, 1992; Durbin and Mitchison, 1990; Obermayer et al., 1990, 1992; Tanaka, 1990; Miyashita and Tanaka, 1992; Grossberg and Olson, 1994) and recently, initial belief that primary response to static oriented lines in the visual field forms the basis of OP maps has been undermined in two ways:

Firstly, in large species particularly, maps of OP appear in the cortex prior to visual experience (Wiesel and Hubel, 1974; Blakemore and Van Sluyters, 1975; Sherk and Stryker, 1976). This problem has been addressed by arguments for the normal occurrence of line-like structure in ante-natal retinal input (Albert et al., 2008; Ringach, 2007; Paik and Ringach, 2011). In contrast to all the above models, Kang et al. (2003) have proposed a model which breaks with the traditional dependence on the primacy of lines, and depends instead on time-invariant correlations in cortical “Mexican Hat” inhibitory surrounds. This model accounts successfully for the apparent isotropy of local intracortical connections and the observed uniformity of sharpness of definition of OP independent of proximity to singularities, and provides a mechanism which might plausibly operate before eye-opening. It requires instead, that LGM inputs to cortex become tuned according to orientation. A further model avoiding the problem of ascription of OP as a primary, stimulus dependent property, explains the conjoint development of OP and ocular dominance columns as a consequence of Hebbian connection formation driven by correlation of visual inputs as a declining function of retinotopic distance of separation at short distances, and reversed correlation of activity in ON and OFF V1 simple cells at greater distances (Erwin and Miller, 1998). All these models however, result in the emergence of OP as a property of line orientation alone, rather than as one attribute of some more complex mechanism of feature response.

Secondly, and more recently, Basole and colleagues, who tested OP using stimulus lines moving at different speeds, and oriented at differing angles to the line of movement of the stimulus, found OP to be a function of these variables to such a degree that for lines oriented non-orthogonally to the direction of movement, OP could vary progressively with increments of speed to an asymptotic limit of 90° (Basole et al., 2003, 2006). Longer lines showed less variation of OP with increasing speed. This finding challenged all models which depended on OP being a fixed “feature” of cortical response, whether or not direct visual stimuli was required to prime the process of self-organization. Basole and colleagues at first concluded that the primal stimulus characteristics are not isolated features such as orientation, direction and speed, but a single characteristic—the “spatio-temporal energy”—that is, the combined spatial and temporal Fourier components of the moving visual stimulus' projection to V1. Subsequent workers explained these results by retaining OP as a primary characteristic, and adding separate consideration of the temporal and spatial frequencies associated with the moving stimuli (Baker and Issa, 2005; Mante and Carandini, 2005; Basole et al., 2006). This analysis was consistent with earlier single unit results, in which tuning of V1 neurons to spatial and temporal frequencies was demonstrated (DeAngelis et al., 1993). Issa and colleagues (Baker and Issa, 2005; Issa et al., 2008) reported that a total of six parameters are required to explain response maps—OP, SF preference, and temporal frequency preference, and the tuning bandwidths of all three. This account is referred to as the spatio-temporal filter model. How these response characteristics arise during cortical development and how neurons become tuned to just those features is the subject of continuing research (Rosenberg et al., 2010), and of this paper.

In common with the model of Erwin and Miller (1998) and that of Kang et al. (2003) the model reviewed here depends upon time-average correlations—that is, the common occurrence of synchronous oscillation in the cortex—although it does not share their other assumptions or conclusions. It seeks to avoid the ascription of “features” as primary characteristics, and to explain both the findings of Basole et al. (2003) and the empirical reduction to alternative feature attributes used in the spatio-temporal model, as well as explaining the emergence of the anatomical features described above.

## Description of model

### Neural field equations

As alternatives to neural network models, lumped neural models and neural field equations have been expressed in many forms (e.g., Wilson and Cowan, 1973; Freeman, 1975; Haken, 1996; Amari, 1977; Nunez, 1981; van Rotterdam et al., 1982; Jirsa and Haken, 1996; Robinson et al., 2001; Wright et al., 2003; beim Graben, 2008; Bressloff, 2012). These offer means of approximating the properties of ensembles of cells on a larger scale then neural networks *per se*. Here we have used a generic form of neural field equations to represent an idealized, isotropic, neural field, representing the developing cortex as if it were not subject to apoptosis—a potentiality from which connections are selected during development. The scale of the field is that of a cortical area such as V1, representing intracortical connections rather than cortico-cortical. Thus, the density of connection between neurons declines with increasing separation of their cell bodies (Braitenberg and Schüz, 1991). The high non-linearity of synapto-dendritic summations are linearized at the field level, and axonal conduction speed is considered single-valued. Subject to these strictures, the following equations include features relevant to the present context:

(1) $$\varphi_{p}^{qr^{\prime }}(t)=f_{p}^{qr^{\prime }}\times Q_{p}\left(r^{\prime },t-\frac{\left|q-r^{\prime }\right|}{\nu }\right)$$

(2) $$\psi_{p}^{qr^{\prime }}(t)=M_{p}^{qr^{\prime }}*\varphi_{p}^{qr^{\prime }}(t)$$

(3) $$\Psi_{p}(q,t)=\int_{r^{\prime }}\psi_{p}^{qr^{\prime }}(t)dr^{\prime }$$

(4) $$V_{p}(q,t)=\sum_{p=e\wedge p=i}G_{p}*\Psi_{p}(q,t)$$

(5) $$Q_{p}(q,t)=f_{\Sigma }(V_{p}(q,t))+E_{p}(q,t)$$

Subscript *p* = *e*, *i* refers to excitatory or inhibitory neurons; superscript **qr**′ refers to synaptic connection from **r**′ to **q** where **q**, **r**′ are cortical positions occupied by single neurons.

ϕ^**qr′**^_*p*_ (*t*) is the flux of pulses reaching pre-synapses at the neuron at **q**, from the neuron at **r**′.

ψ^**qr′**^_*p*_ (*t*)is the synaptic current generated by ϕ^**qr′**^_*p*_ (*t*).

Ψ_*p*_ (**q**, *t*) is the aggregate synaptic current of type *p* generated at **q**.

*V*_*p*_ (**q**, *t*) is the soma membrane potential (relative to the resting potential) generated at **q**.

*Q*_*p*_ (**q**, *t*) is the pulse emission rate at **q**.

*f*^**qr′**^_*p*_ is the probability density of occurrence of pre-synapses generated by axons of the neuron at **r**′ terminating at **q**.

ν is axonal conduction speed.

*M*^**qr′**^_*p*_ is the steady-state term in a convolution transforming pre-synaptic flux to synaptic current.

*G*_*p*_ is the steady-state term in a convolution transforming pre-synaptic flux into dendritic potentials.

*f*_Σ_ (*V*_*q*_ (**q**, *t*)) is a sigmoid function describing the local conversion of dendritic potentials into the rate of generation of action potentials.

*E*_*p*_ (**q**, *t*) is a driving signal noise, arising from intrinsic random cell action potentials.

Restriction of the field to the scale of a cortical area carries several implications important for the model, all because the probability of connections between any two neurons declines with distance of separation. Firstly, descriptively we can consider “reciprocal couplings” as an idealization/representation of field coupling symmetry, and in many instances reciprocal couplings will in fact exist. Secondly because of more generally dense connections among near neighbors, smoothing at dendritic summation requires that *Q*_*p*_ (**q**, *t*) is spatially and temporally “brown”—i.e., has high correlation at short distances and times of separation. Thirdly, the average “degree” of separation—i.e., the average number of neighboring cells traversed by synaptic connections linking one cell to another—will also increase in proportion to physical distance of separation.

Experimental observations (Freeman, 1975, 1991; Hassenstaub et al., 2005) show intrinsic cortical oscillation arises from alternating excitatory cell and inhibitory cell firing at lags ¼ of the period of oscillation. Simulations of the oscillations (Wright, 2009, 2010) show that traveling waves are thus generated, the intersection of which produces broadband synchrony. In conditions of uniform cortical excitation without strong perturbation from external inputs the exchange of pulses between all cells reaches an equilibrium—that is, a steady-state of symmetrical exchange of signals between excitatory cells at any two positions on the cortex, so that over sufficient intervals, *T*,

(6) $$\frac{1}{T}\int_{T}\left(\varphi_{p1}(q)-{\bar{\varphi }}_{p1}\right)dt=\frac{1}{T}\int_{T}\left(\varphi_{p2}(r^{\prime })-{\bar{\varphi }}_{p2}\right)dt$$

where ${\bar{\varphi }}_{p}$ is the time-average presynaptic flux, uniform throughout the cortical field. The equilibrium reached implies differences in timing between the firing of excitatory and inhibitory cells. The interaction of excitatory and inhibitory cells (*p*1 ∨ *p*2 = *e*, and *p*1 ∨ *p*2 = *i*,) leads to closely correlated firing of both cells if they are very closely situated, as a consequence the similar local values of *E*(**q**, *t*) equation (5), while ¼-cycle-out-of-phase oscillation develops between more separated excitatory and inhibitory cells. Inhibitory/inhibitory or excitatory/excitatory interactions (*p*1 ∧ *p*2 = *e*, or *p*1 ∧ *p*2 = *i*,) between reciprocally connected neurons lead to zero-lag synchrony, and since conduction delays are short compared to the period of oscillation, the equality of equation (6) is generally approached even when *T* is smaller than the period of oscillation (Chapman et al., 2002). As there are equal time-lags in both directions of conduction excitatory pulse trains throughout the cortex have maximum correlation at zero lag—i.e., where ${\bar{Q}}_{e}$ is the time-average firing rate—also uniform throughout the cortical field -

(7) $$\left(Q_{e}-{\bar{Q}}_{e})(r^{\prime },t)\approx (Q_{e}-{\bar{Q}}_{e})(q,t\right)$$

**Figure 1:**
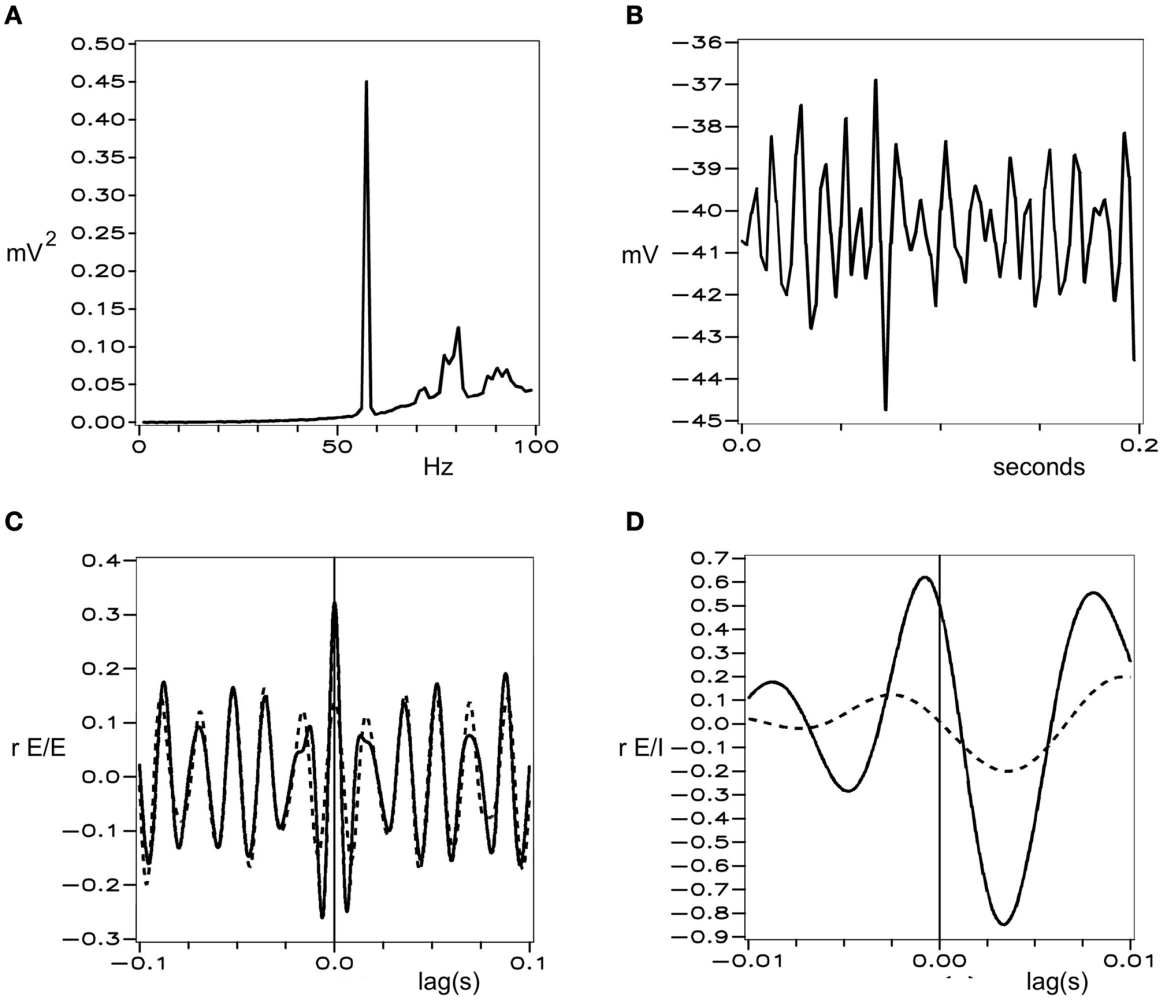
**Simulated electrocortical activity in the excited cortex, from Wright (2010). (A)** Power spectrum of a local field potential time-series, shown in **(B)**. **(C)** and **(D)** Cell firing correlations, vs. time-lag. Dashed line—cells remote from each other. Solid lines—cells adjacent to each other. **(C)** Between excitatory cells. **(D)** Between excitatory and inhibitory cells.

**Figure 2:**
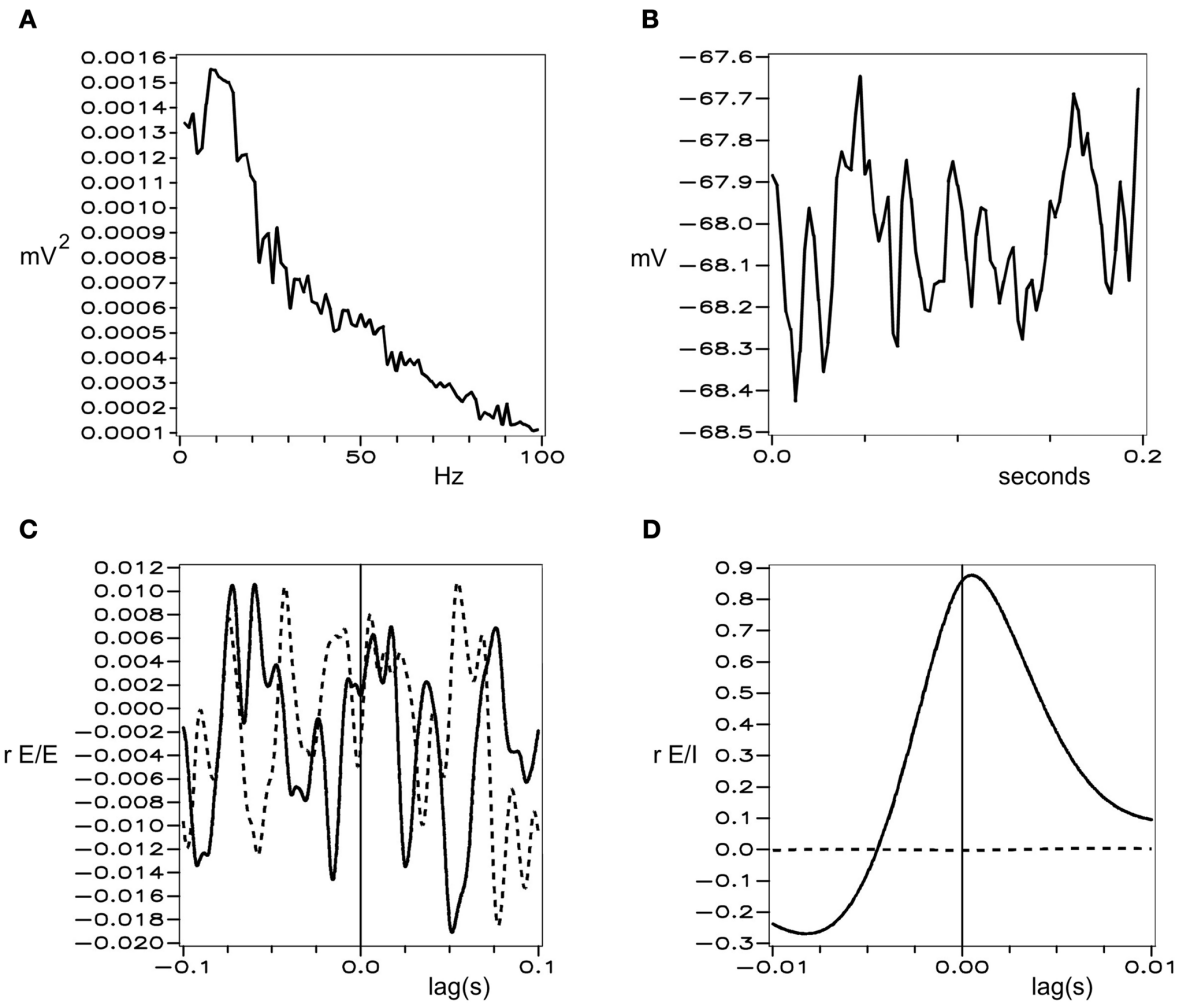
**Simulated background electrocortical activity, in conditions of low cortical excitation.** Graphical format is the same as in Figure 1.

Figures 1 and 2 show these properties generated in a simulation of cortical dynamics with physiologically realistic parameters (Wright, 2009, 2010). In conditions of strong cortical excitation local oscillation is autonomous and corresponds to cortical gamma rhythm, while in conditions of lower cortical excitation, damped gamma oscillation, and a predominance of background 1/*f*^2^ is seen.

### Magnitude of pre-synaptic pulse synchrony

Zero-lag synchronous oscillation thus entails presynaptic pulse synchrony, with a magnitude of presynaptic flux variation which can be defined respectively for individual synapses, individual cells, and in aggregate, as

(8) $$J^{qr^{\prime }}={\left[\frac{1}{T}\int_{T}{\left(\varphi_{e}^{qr^{\prime }}-{\bar{\varphi }}_{e}\right)}^{2}dt\right]}^{1/2}$$

(9) $$J^{q}={\left[\frac{1}{T}{\int_{T}\int_{r^{\prime }}\left(\varphi_{e}^{qr^{\prime }}-{\bar{\varphi }}_{e}\right)}^{2}dtdr^{\prime }\right]}^{1/2}$$

(10) $$J={\left[\frac{1}{T}\int_{T}\int_{r^{\prime }}\int_{q}{\left(\varphi_{e}^{qr^{\prime }}-{\bar{\varphi }}_{e}\right)}^{2}dtdr^{\prime }dq\right]}^{1/2}$$

*J*^**qr′**^ is RMS presynaptic flux variation between **q** and **r**′, *J*^**q**^ is the sum of *J*^**qr′**^ at a single excitatory neuron, and *J* is the aggregate of *J*^**q**^ over the cortex.

### Selection of scale-free small-world configurations of neurons

For any given level of cortical excitation, *J* is greatest for that ensemble of *C* connected neurons, in which excitatory pulses arrive at dendrites, from all sources at differing distances of separation, as closely in-phase as possible, so as to maximize their summation. Axonal delays, small compared to the period of gamma oscillation, contribute a phase difference between cell firing at **r**′ and the arrival of presynaptic pulses at **q**, of

(11) $$\Delta \Phi^{qr^{\prime }}=2\pi \frac{\left|q-r^{\prime }\right|}{P\nu }$$

where *P* is the period of oscillation. Therefore that ensemble selected by its capacity to maximize presynaptic synchrony must approach minimal total axonal length, $L=\int_{r^{\prime }}\int_{q}\left|q-r^{\prime }\right|dqdr^{\prime }$, and minimization of this length minimizes the metabolic requirements of the axons.

It has been shown generally (Cohen and Havlin, 2003) for all systems of connected elements, the path length in a topological sense is at a minimum where degree distribution follows a power law. As was pointed out in conjunction with equations (1–5), in our idealized neural field, average degree of separation, in the topological sense, increases linearly as metric distance of separation of the cell bodies, so that if *L*, their total length of axonal connections, is minimal, then the path length in the topological sense is also minimal, and the degree distribution is that of a scale-free, or ultra-small world. Therefore, the connection density between cells vs. their metric distance of separation should also be approximated by a power-law distribution. Further, according to Cohen and Havlin

(12) L~loglogC

so the metabolic efficiency of the connection system is further enhanced if the surviving cells are linked into a continuum, as opposed to separate pools of neurons.

In accord with equation (1), the number of neighboring excitatory cells connected to a given excitatory neuron, as a function of distance of separation, is proportional to 2 π × *f*^**qr′**^_*e*_ (|**q** − **r**′|)—so the ensemble of neurons selected by greatest synchrony must have a connection density function of the form:

(13) $$f_{e}^{qr^{\prime }}\sim {\left(2\pi \left|q-r^{\prime }\right|\right)}^{-A}\;A>0$$

Intracortical axonal trees have approximately exponential density/range relations (Scholl, 1956; Braitenberg and Schüz, 1991) and a power function is fitted exactly by an infinite sum of exponential functions—i.e.:

(14) $${\left(2\pi \left|q-r^{\prime }\right|\right)}^{-A}=\frac{1}{\Gamma (A)}\int_{0}^{\infty }u^{A-1}exp\left[-u2\pi \left|q-r^{\prime }\right|\right]du$$

so an ultra-small-world connectivity can be achieved by sets of populations of cells with differing axonal characteristic lengths. During embryogenesis primal cells divide sequentially by layer (Rakic, 1988; Shi et al., 2012) with differences in growth pattern and characteristic axonal length programmed in sequential cell divisions. For simplicity, we consider only two populations of excitatory cells, with cell bodies partially separated by layer, but with intermingled axonal and dendritic trees, and axonal tree connection probabilities described by:

(15) $$f_{\alpha }^{qr\prime }=\frac{N_{\alpha }}{N}2\pi \lambda_{\alpha }\text{exp}\left[-\lambda_{\alpha }2\pi \left|q-R\right|\right]$$

(16) $$\begin{array}{l} f_{\beta }^{\text{qr′}}=\frac{N_{\beta }}{N}2\pi \lambda_{\beta }\text{exp}\left[-\lambda_{\beta }2\pi \left|q-r\right|\right]\\ f_{e}^{qr^{\prime }}=f_{\alpha }^{qR}+f_{\beta }^{qr} \end{array}$$

*f*^**qR**^_α_ refers to the axonal trees with longest axonal extensions, and *f*^**qr**^_β_ refers to the axonal trees with short axonal extension, thus λ_α_ < λ_β_. *N* = *N*_α_ + *N*_β_ is the number of synapses received/generated by each cell. Distances from **r**′ to **q** are substituted as **r**, **R** to indicate equal distances, |**q** − **r**| and |**q** − **R**|, measured along the axonal trees of the respective populations.

The further defining characteristic of small-world connectivity—the occurrence of connection nodes—emerges as a consequence of the formation of the superficial patch system, as follows.

### The origin of the superficial patch system

The two populations of cells described by equations (15) and (16), and the synapses they give rise to can be referred to as α-cells and synapses, and β-cells and synapses. We first make a provisional assumption (later justified on a species-specific basis) that *N*_β_ >>> *N*_α_, so that α-cells with long-range axons are embedded among much more numerous β-cells, all with sparse connectivity. Equation (10) can be written by separately summing contributions from α-cells at positions {**q**α} and β-cells at positions {**q**β}, to give:

(17) $$J=\int_{q\alpha }\int_{R}J^{qR}dq\alpha dR+\int_{q\beta }\int_{r}J^{qr}dq\beta dr$$

so *J* is at a maximum if ∫_**q**α_∫_**R**_*J*^**qR**^*d***q**α*d***R** and ∫_**q**_β∫_**r**_*J*^**qr**^*d***q**β*d***r** are individually at maxima. Applying equations (15) and (16) via equation (1) to find values of *J*^**qr′**^ in equation (8) as functions of |**q** − **r**, **R**|, shows that:

(18) $$\begin{array}{l} J^{qr}=J^{qR}\text{ if }\left|q-r,R\right|=x\\ J^{qr}>J^{qR}\text{ if }\left|q-r,R\right|<x\\ J^{qr}<J^{qR}\text{ if }\left|q-r,R\right|>x \end{array}$$

where $x=-ln\left(\frac{N_{\alpha }\lambda_{\alpha }}{N_{\beta }\lambda_{\beta }}\right)/2\pi (\lambda_{\beta }-\lambda_{\alpha })$

Consequently ∫_**q**_β∫_**r**_*J*^**qr**^*d***q**β*d***r** is at a maximum if β-cells are clustered so they make reciprocal connections at minimum distance and maximum density (β-clusters). β-cells at the center of β-clusters, for which *J*^**qr**^ attains the maximum possible value, must give and receive all their connections as β-connections to a radial distance of *x*.

Since β-cells are clustered, α-cells necessarily are also clustered (α-clusters), and since maximization of reciprocal β-connections excludes formation of short-range reciprocal α- connections, α-cells must form reciprocal synaptic connections at distances greater than *x*, to maximize ∫_**q**α_∫_**R**_*J*^**qR**^*d***q**α*d***R**. Similarly, reciprocal connections between α- and β-cells must occur at cluster margins, over distances approximate to *x*. Since we made the provisional assumption that *N*_β_ >>> *N*_α_, then fitting the sum of equations (15) and (16) to a power function requires λ_α_ <<< λ_β_. Consequently α-cells may form multiple patches of synaptic connections, skipping from α-cluster to α-cluster.

Since β-clusters have radius *x* and α-clusters are separated by distance *x*, α-clusters are necessarily placed at the vertices of hexagons tiling the cortical surface, with each hexagon embracing a β-cluster. Analogy to the superficial patch system in some species is apparent.

As noted earlier, hexagonal symmetry of OP and the superficial patch system is an idealization that is roughly approached in some species, while in others it is effectively absent (Horton and Adams, 2005). Since approximation of a power law distribution by two populations of neurons requires *N*_α_ <<< *N*_β_ if λ_α_ <<< λ_β_, this case is more closely approached for larger cortical sizes, and the patchy connection system will have higher orderliness and hexagonal rotational symmetry. If λ_α_ < λ_β_ by only a small amount, as in animals with small cortical size, then *N*_β_ is not necessarily greater than *N*_α_, and an ordered hexagonal structure need not be apparent. Such reduction of the apparent orderliness does not imply the absence of “small world” connectivity, nor imply impairment of function. The comparative invariance of distance between OP singularities across species reported by Kaschube et al. (2010) and Keil et al. (2012) implies that $x=-ln\left(\frac{N_{\alpha }\lambda_{\alpha }}{N_{\beta }\lambda_{\beta }}\right)/2\pi (\lambda_{\beta }-\lambda_{\alpha })$ [equation (18)] is also relatively constant over species in the middle to large range of V1 size. Since the ratios *N*_β_/*N*_α_, and λ_α_/λ_β_ must vary inversely in value in different species, according to cortical size, as required if the sum of the two synaptic distributions maintains a power law distribution, then comparative invariance of OP singularity density is to be expected.

### Local synaptic competition for metabolic supply

Turning from optimization of energy demand of axons, to that of dendrites, we can modify equation (2) to a form representing complex distinct processes of synaptic adaptation, impulse decay, and pre-synaptic synergy, including the limiting rate of metabolic energy supplied to excitatory synapses—viz:

(19) $$\psi_{e}^{qr^{\prime }}(t)=\Gamma^{qr^{\prime }}M_{e}^{qr^{\prime }}*\varphi_{e}^{qr^{\prime }}(t)$$

(20) $$M_{e}^{qr^{\prime }}=D\times S$$

Γ^**qr′**^ is the available fraction of the metabolic supply rate needed to attain maximum current flow. Since we have assumed increasing synaptic current in synchronously activated synapses increases the available metabolic supply, the value of Γ^**qr′**^ must follow that of ψ^**qr′**^_*e*_.

(21) $$D=\frac{1}{B}\exp[-Bt]\;B>0$$

represents impulse decay following delivery of an afferent action potential, with time-integral of 1 (after Rennie et al., 2000).

(22) $$S=1/(1+\exp[-g(J^{q}(t))]$$

is a sigmoid function with range 0–1, representing synaptic adaptation to the afferent pulse rate, and including the effect of pre-synaptic co-operation (Tsukada and Fukushima, 2010) upon individual synaptic current flow as *g*(*J*^**q**^)—a suitable ascending function in *J*^**q**^, such that if *J*^**q**^ = 0, there is no current flow at the synapse.

As well as inter-cellular competition between assemblies of neurons, we assume competition takes place between adjacent individual synapses arising from the same neuron. Therefore those neurons that survive apoptosis must have found an efficient deployment of resource to the synapses best positioned to maximize the magnitude of synchrony. Any two adjacent synapses arising from the same pre-synaptic neuron may terminate on the same, or different, post-synaptic neurons. If they terminate on the same neuron their conditions are essentially identical in terms of equations (19–22). If they terminate on different neurons, then the relevant values of *J*^**q**^ need not identical—and their competition for resources would lead, via the feedback between ψ^**qr′**^_*e*_ and Γ^**qr′**^, to low synaptic current at one synapse, and high current at the other. Just what the physiological corollary of these opposite high and low-activity states is, and the critical metabolic component for which the synapses compete, we do not specify. A likely, but by no means unique contributing factor is the supply of extracellular calcium (Montague, 1996). Whatever the critical component(s), the important consequence is that, at synchronous equilibrium, closely situated neurons have either high, or low, pulse correlations with each other.

### Organization of pre-vision response properties

**Figure 3:**
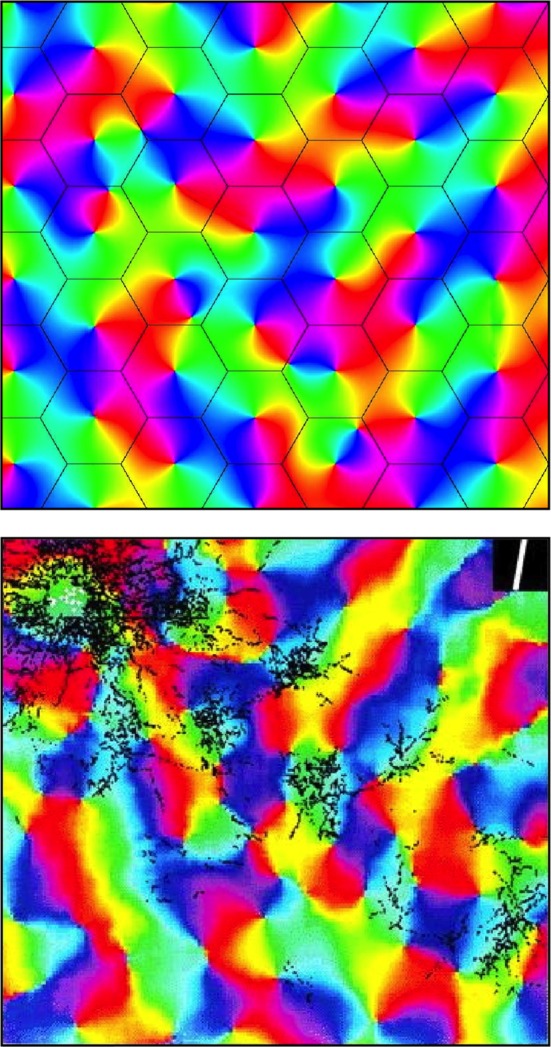
**Simulated and real maps of orientation preference in V1, from Wright et al. (2006). Top:** Simulation. Colors of the spectrum, from red to violet, represent average OP of V1 neurons for slow-moving visual lines of orientation 0 − π. Adjacent macrocolumns, of diameter ~300 μm are set within a hexagonal frame (the patch system) with OP forming color wheels about OP singularities. Orientations and chiralities of the color wheels are arranged to approach a minimum total of angular disparity from mirror reflection of OP between each macrocolumn and its neighbors. **Bottom:** Real OP. Visualized in the tree shrew by Bosking et al. (1997). Superficial patchy connections are demarcated in black by a selective stain. Scale of macrocolumns is approximate to that of the simulation.

We can now term those synapses that are transmitting impulses more strongly near equilibrium “saturated” synapses, and those which are more quiescent, but potentially able to be activated, “sensitive” synapses, and can consider what spatial patterns of saturated connections would best meet the requirement to maximize synchrony. Here a further property of the neural field commented on in relation to equations (1–5)—higher spatial cross-correlation of pulses and field potentials at shorter range — has a decisive impact on the equilibrium pattern of synaptic saturations. These emergent patterns, diagrammed in Figure 3, arise for the following reasons:

- Maximum synchrony generation with highest cross-correlation among near-neighbors in each β-cluster requires saturated couplings link near-neighbor cells—but sensitive connections must also form between closely adjacent β-cells. Both requirements are met when saturated connections within each β-cluster form a re-entrant network analogous to a Möbius strip. A similar argument regarding connections formed within macrocolumns has been advanced earlier (Wright et al., 2006; Wright and Bourke, 2008).
- The α-cluster system and each of the β-clusters must enter into maximum joint resonance. This requires the formation of a homeomorphic projection between scales. The projection must be homeomorphic, since spatial cross-correlation is constrained to decline with distance at both scales, and so if resonance is at a maximum, the projection map must be one preserving topological identity between scales. This is possible because a disk can be mapped to a Möbius strip. Thus saturated α-cell to β-cell synapses must systematically map limited angular ranges of the surrounding α-system onto limited angular ranges on the margins of each β-cluster, and receive reciprocal saturated β-cell to α-cell synapses. Such a mapping requires specification of an orientation and chirality for each β-cluster, and requires a reciprocal distribution of saturated and sensitive synapses from opposite sides of the α-system to neurons in a limited angular range within each β-cluster.
- Maximum synchrony generation with high cross-correlation among near-neighbors in the α-system requires α-cells be linked by saturated synapses. This requirement is concordant with deployment of the excess sensitive α-connections to neurons in β-clusters at positions outside the homeomorphic projection.
- Saturated and sensitive β − β connections between adjacent β-clusters must also be arranged to maximize resonance. Therefore β-clusters must project to each of their six neighbors as closely as possible to mirror symmetry, with both saturated and sensitive synapses linking homologous points—that is to say, points with similar OP as classically measured with low object speeds—within each cluster. Perfect mirror symmetry is not possible between all adjacent clusters within a hexagonal array, so mirror-symmetry can be only approximate and irregular and the necessarily broken symmetry permits the particular pattern generated to be one of a large set of possible combinations.

Further analogy between the hypothetical α- and β-systems and real anatomical structures can now be drawn. As well as the α-system's congruence with the superficial patch system, the β-systems, each with a dense system of local connections that are centrally spared from patchy connections, are analogous to macrocolumns each centred about an OP singularity. The distribution of OP for lines of orientation 0 − π to angles 0 − 2π in pinwheels about a singularity finds analogy in the wrapping of a Euclidean plane onto a Möbius strip. It has also been earlier shown that arrangements of adjacent pinwheels in broken mirror symmetry match classical OP maps (Wright et al., 2006).

**Figure 4:**
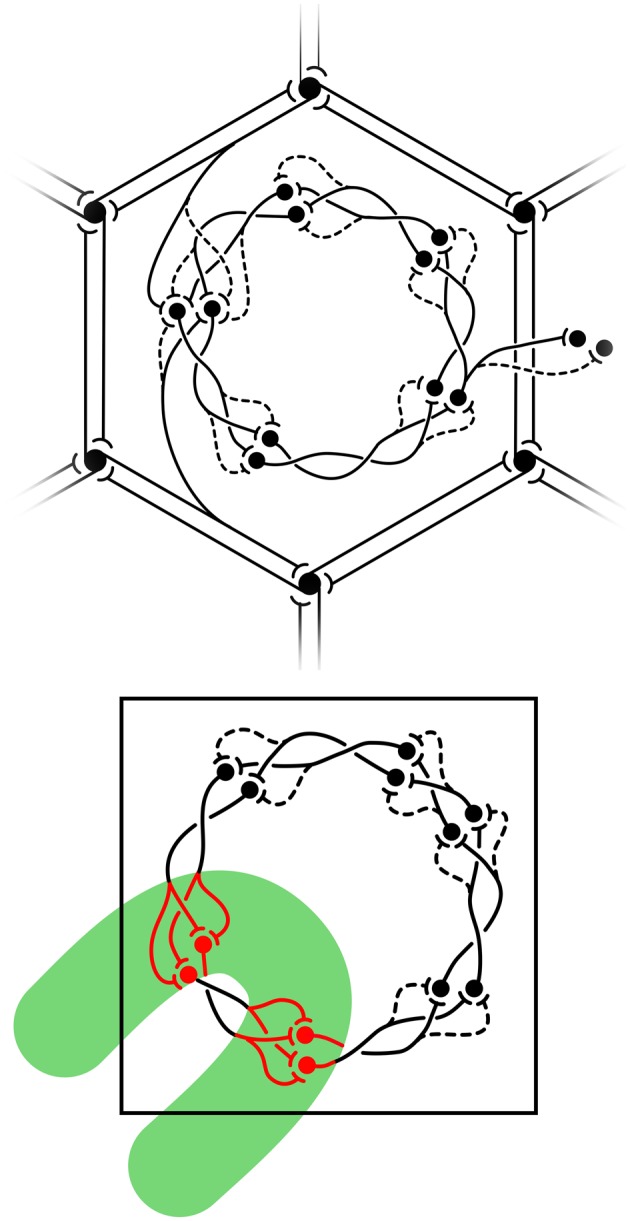
**Equilibrium distribution of synaptic activity, and the impact of visual inputs disrupting equilibrium from Wright and Bourke (2013). Top**: equilibrium disposition of saturated and sensitive synapses. Black circles represent cell bodies and dendrites. Synapses are indicated as saturated (solid) or sensitive (dashed) terminations of axons. Reciprocal connections between α-patches (patchy connections) form the hexagonal array. (Other connections, although shown as unidirectional, are also reciprocal.) A representative pair of connections from α-cells to the β-patch is displayed in the upper-and lower-aspects of the figure. At the center of the figure, saturated and sensitive synapses show the network's analogy to a Möbius-strip within a β-patch (macrocolumn). To the right, representative links from the central macrocolumn to cells at homologous positions in neighboring macrocolumns. **Bottom**: exposed to strong transient signals conveyed over the superficial patch system, summing with direct visual inputs conveyed to the cRF, the equilibrium configuration breaks down. The green bar represents the field of excitation of cells by the contextual signals, within which cells also directly excited in the cRF, fire at high rates.

The structure of real patchy connections and classical OP response maps, contrasted with the results of simulating the arrangement of adjacent macrocolumnar structures in accord with the description above, are shown in Figure 3, while Figure 4 shows diagrammatically the proposed arrangement of saturated and sensitive synapses, and foreshadows the effect of structured visual stimuli, once the post-natal phase of development begins—to be described in the next section.

**Figure 5:**
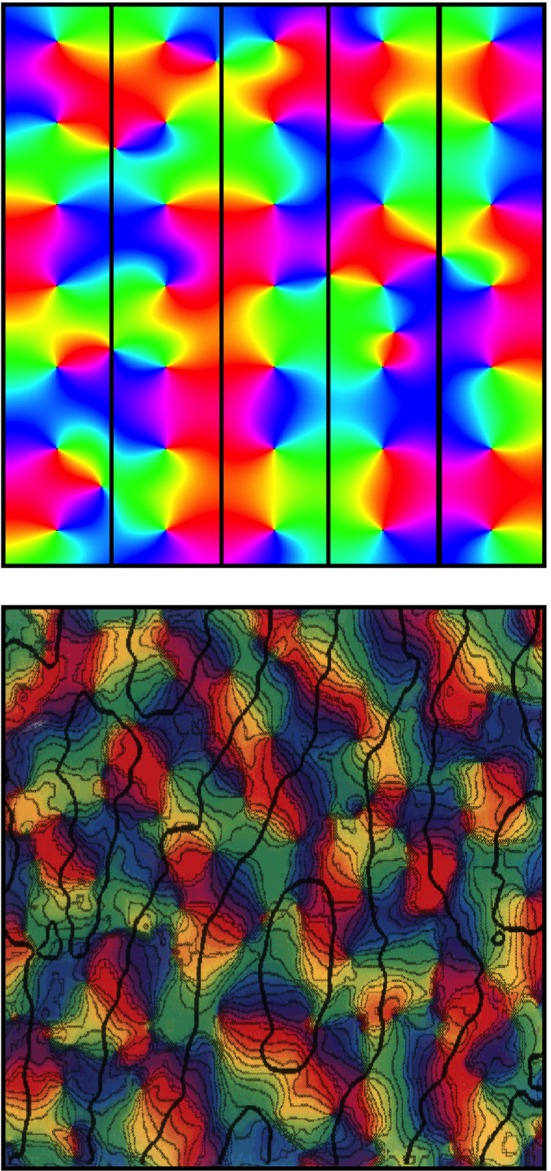
**Top:** Simulation of OD columns in accord with Wright and Bourke (2008). **Bottom:** Real OD columns, visualized by Obermayer and Blasdel (1993). Color coding of OP and scale as for Figure 3. Black lines demarcate alternation of OD between columns. Fine black lines in the lower figure trace the way OP is aligned so it matches orthogonally across OD column boundary.

Figure 5 shows a further impact upon response map organization—the emergence of OD columns.

Just as OP organization in some species is apparent before eye opening, so too is the organization into OD columns (Blakemore and Van Sluyters, 1975; Erwin and Miller, 1998). Explanation of this can be included in the present model by an argument similar to that of Erwin and Miller, who suppose the correlation of cell firing at short distances of separation of V1 cells to be greater than the correlation of visual inputs over a similar distance. This forces a columnar OD organization because of instability—in the present model's terms, the resulting disruption of the synchronous field at equilibrium produced by binocular inputs to the same cells—resolved by formation of columns in Turing patterns. A corollary of this effect is impact on the hexagonal arrangement, with broken mirror symmetry of OP organization, predicted in (d) above. The required alternation of OD columns would imposes a frustration on the approach to hexagonal tiling of the cortical surface—forcing approach closer to a square tiling. The occurrence of mirror symmetry within a square tiling accounts for the way that lines of OP cross orthogonally between OP columns. (Obermayer and Blasdel, 1993). Following eyeopening inputs from the two eyes transmit images which are necessarily cross-correlated at a small spatial lag, because of angular disparity in their line of focus. Spatial lag correlation in their inputs at V1 level could then help maintain the columnar organization (Wright and Bourke, 2008).

### Wave transmission of visual information, following eye-opening

We compactly express the emergent map by which the patchy connections over a part of V1 link to positions within each macrocolumn, as an homeomorphic projection from a disk on a Euclidean plane, **P**, to a Möbius strip, **p**^[2]^ - the square brackets [2] indicating the map's resemblance, if viewed from a third dimension, to a 2:1 map formed by squaring a complex vector. Defined in polar co-ordinates,

(23) $$P\left(\left|R-C_{j}\right|\text{​},\vartheta \right)\to p^{\left[2\right]}\left(\left|r-C_{j}\right|,\pm \vartheta +\varphi \right)$$

where **C**_*j*_ is the origin of both **P** and **p**^[2]^ for the *j* − *th* local map, and corresponds to the position of the OP singularity in that macrocolumn. ϑ is the polar angle of **R**, chirality of the local map is indicated by ± ϑ, and ϕ is the orientation of the local map relative to the global map. ϑ + ϕ is defined on the range 0 − 2π in both local and global maps, but is represented with apparent angle doubling in the local map. This describes a topology for “contextual” connections (Li et al., 2000; Angelucci and Bullier, 2003) to each macrocolumn.

Visual input after eye opening will cause departures from the equilibrium condition. Let *O*(**P**, *t*) be a visual image projected to V1 by the direct visual pathway. Laterally traveling waves of pulses and local field potentials relayed by the patchy connections can transmit that image to each local map with a point to point delay, $\frac{\left|R-r\right|}{\upsilon }$, where ν now represents wave speed, so that

(24) $$O\left(P,t\right)\to O\left(p^{\left[2\right]},t+\frac{\left|R-r\right|}{\nu }\right)$$

Suppose *O*(**P**, *t*) is a segment of the image of a visual line, traveling with uniform velocity, **V**_*x*_, along an *x*−axis directed toward a macrocolumn with its singularity at **C**_*j*_. *O* has a component of its extension on the *x*−axis, *O*_*x*_, and an orthogonal component of extension, on the *y*−axis, *O*_*y*_. *K*_*x*_ is the dominant spatial frequency of *O*_*x*_, and *K*_*y*_ is the dominant spatial frequency of *O*_*y*_. Then the local map projection of *O* has a transformed spatial frequency in the *x*−axis but not in the *y*−axis—i.e.:

(25) $$k_{x}\propto \frac{\nu }{\nu \pm V_{x}}K_{x}$$

(26) $$k_{y}\propto K_{y}$$

where *k*_*x*_, *k*_*y*_ are the spatial frequencies in the local map projection of *O*, and the sign ± in equation (25) depends on whether *O* is approaching or departing from **C**_*j*_. That is, *O*'s orientation in the global map is transformed to its projection to corresponding areas in the local map, by Doppler shift, with a difference in orientation, δϑ;

(27) $$\delta \vartheta =\left|tan^{-1}[K_{y}/K_{x}]-tan^{-1}[k_{y}/k_{x}]\right|$$

### Interaction of contextual signals and the classic receptive field

**Figure 6:**
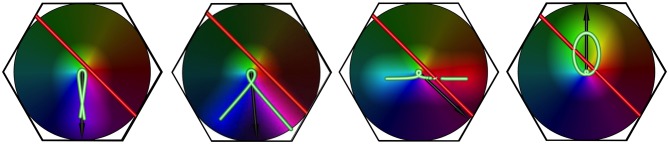
**The effect of increasing stimulus speed on apparent OP, for a bar of length 6 units, oriented at 45° to its direction of motion, and traveling left to right.** Examples shown are freeze-frames, from separate simulation movies, at similar positions in the visual stimulus' transit across the macrocolumn. From left to right, in each example, the bar speed/wave speed is 0.1, 0.5, 1.0, 1.5, respectively.

Laterally transmitted contextual signals generally do not trigger cell firing, until the classic receptive field (cRF) is directly stimulated (Li et al., 2000; Angelucci and Bullier, 2003) via the visual pathway. Those cells that then fire within a macrocolumn are those that reflect the supra-threshold summations of sub-threshold signals conveyed over the contextual, patchy, connections, and the direct pathway. We next assume that the summation of contextual and direct cRF inputs acts as an impulse causing a transient breakdown of equilibrium, during which synapses that were in the sensitive state in equilibrium briefly generate substantial synaptic currents [See Figure 4 (Bottom) and Figure 6]. Action potentials are triggered in surrounding cells, and subsequently there is a restoration toward the equilibrium state on withdrawal of the stimulus. During the breakdown the mapping of activity from the global to the local map becomes:

(28) $$O\left(P,t\right)\to O\left(p^{2},t+\frac{\left|R-r\right|}{\nu }\right)$$

The change from equation (24) made by removal of the square brackets from *p*^[2]^ represents the breakdown's form, as itself a map from global to local scale, resembling a 2:1 complex-multiplication map, as initially described by Alexander et al. (2004). The 2:1 map implies that single cells would show similar responses to a stimulus moving in either direction, but because firing is initiated over contextual connections in a 1:1 mapping, multi-cellular recordings would show that the spatial and temporal order of firing of neurons was unique for a given stimulus form and velocity.

### Post-natal effects of learning, the spatio-temporal filter model, dimension reduction, and “like to like” connections

Equations (2,3,4, 19–22) contain state-variables required by mathematical expressions of physiological versions of the Hebb rule, and the spatio-temporal learning rule (Elliott and Shadbolt, 2002; O'Connor et al., 2005a,b; Enoki et al., 2009; Tsukada and Fukushima, 2010; Elliott, 2011). Following eye opening, stimuli with regularly repeated spatial and temporal structure reach V1, so we assume that exposure to a repeated stimulus leads to permanent synaptic consolidation of connections, overlaying those formed in the ante-natal, equilibrium condition. As remarked in the Introduction, Baker and Issa (2005) have shown that all V1 response features can be described in terms of six variables—optimal values of OP, spatial frequency preference, and temporal frequency preference, each associated with a Gaussian bandwidth of tuning of the cortical response to these features. These define three hypothetical filter proceeses. However, stimulus variables in the present model have equivalents to those used in the spatio-temporal filter model. These are:

**Table:** 

**Spatio-temporal model**	**Present model**
Object orientation	Orientation relative to the y-axis defined for equations (25, 26)
Object velocity	**V**_*x*_
Object drift angle	tan^−1^[*K*_*y*_/*K*_*x*_]
Object spatial frequency	*K*_*x*_/|cos(tan^−1^[*K*_*y*_/*K*_*x*_])|
Object temporal frequency	**V**_*x*_*K*_*x*_

Repeated stimulation with a particular stimulus will therefore lead, under Hebbian learning, to maximization of the response to that stimulus, thus creating an apparent “tuning” of particular neurons to that particular combination of stimulus features. Thus, the spatio-temporal model can be regarded as a consequence of the present model. Optimization by learning of the parameters for each of the three filters must be competitive between adjacent cells, providing the necessary condition for fitting response maps with continuity and completeness, by dimension-reduction methods (Kohonen, 1982; Durbin and Willshaw, 1987; Durbin and Mitchison, 1990).

Finally, the consolidation of saturated long-range patchy connections by Hebbian learning would result in mature “like to like” connections.

## Simulations—a critical test

A critical test of our model, then, is whether we can reproduce in simulation the results of Basole et al. (2003), without *a priori* feature-specific responses to orientation, spatial frequency, or temporal frequency. Our simulations assume the steady-state presence of the Mobius synaptic configuration and its perturbation by visual signals, intended to reflect the state of the visual cortex shortly after birth, when first exposed to visual stimuli.

Equation (28) was applied in simulations of an hexagonal array of seven adjacent macrocolumns. Results reported here are for the central macrocolumn of the array of 7. Examples are shown in Figure 6, which shows the orthogonal transformation of apparent OP from the lowest to the highest bar speed for a moving line stimulus oriented at 45° to its line of passage.

Diameter for each macrocolumn is 300 microns, and wave speed for transcortical polysynaptic propagation 0.1 m/s (Bringuier et al., 1999). Units of length subsequently referred to, are multiples of the radius of a macrocolumn—150 microns. Simulation time-step was 0.1 ms.

A moving line in the visual field, relayed by the direct visual pathway to the cRF of each macrocolumn is represented as a red bar. In a single simulation the red bar traveled across the entire hexagonal array from left to right, with constant speed, direction and orientation. The orientation of the red bar to the line of passage is measured as *bar angle* from 0°, where the bar is oriented orthogonally to the direction of travel, to ±90°, where the bar is oriented in the direction of travel.

The lag-transmitted image of the red bar, relayed as subthreshold activation to each macrocolumn via the superficial patch system, is shown in green, with illumination about the zone of subthreshold activation, to indicate that input to the cRF from the direct visual pathway and contextual signals caused triggering of action potentials. The average angle from the macrocolumn singularity to the centers of action potential generation (i.e., all points on the green line with illumination) was calculated at each time-step, and shown as a black arrow, thus indicating the part of the macrocolumn with a response preference (*apparent OP*) for the particular bar movement. (A change in the sector of the macrocolumn that is maximally stimulated is equivalent to an equal change in the angle of approach of the bar needed to maintain stimulation of the same sector). The black arrow angle was averaged over a window beginning after the red bar had passed the center of the macrocolumn by a distance equal to 10% of macrocolumn radius, and extending from the 10th percentile to the 20th percentile of that radius, thus obtaining an estimate of the apparent OP during the cRF activation time. The standard error (SE) of the black arrow angles was calculated from 11 equally spaced time steps through the averaging window.

Combinations of bar-length, orientation of the bar to the direction of movement, and bar speed, were then systematically varied in separate simulations, results of which are supplied as supplementary animated movies. Their effects on OP, measured at the central local map of the hexagonal group, were obtained as *OP difference*, **Δϕ**—a measure of the change in OP as a function of these variables—calculated as

(29) $$\Delta \phi =\{\begin{array}{c} \phi_{1}-\phi_{0}-\pi \\ \phi_{1}-\phi_{0}\\ \phi_{1}-\phi_{0}+\pi \end{array}\;\begin{array}{c} \text{when}\\ \text{when}\\ \text{when} \end{array}\;\begin{array}{c} \pi /2<\phi_{1}-\phi_{0}\\ -\pi /2\leq \phi_{1}-\phi_{0}\leq \pi /2\\ \phi_{1}-\phi_{0}<-\pi /2 \end{array}$$

The *reference OP*, **ϕ**_0_ ∈ [0, π), was the OP found at the lowest bar speed applied (bar speed/wave speed = 0.1) and the *apparent OP*, **ϕ**_1_ ∈ [0, π), was the OP found at higher speeds.

**Figure 7:**
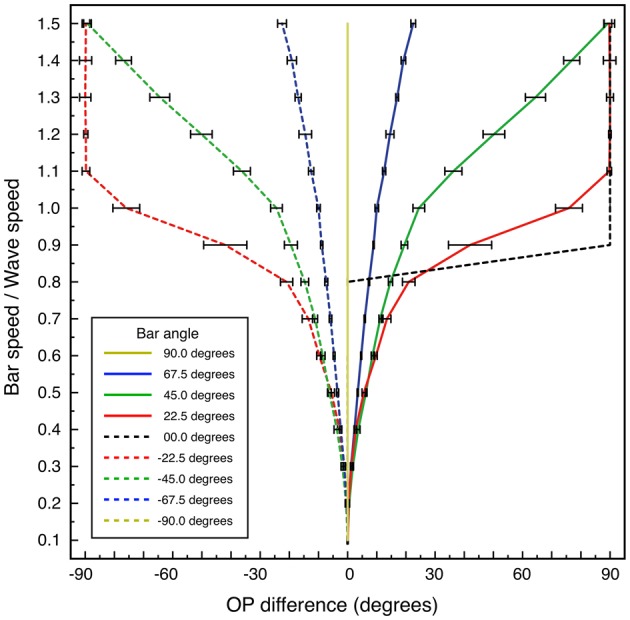
**Change in apparent OP, and standard error of the estimate, as a function of bar speed to wave speed, for lines at different orientations to their directions of motion.** Bar length 6 units.

Systematic results are shown in Figure 7, which graphs OP difference vs. bar speed/wave speed, for bar angles 0 to ±90°, calculated for a bar length of 6 units. Variation of bar length showed progressive lessening of the effect of velocity on OP for greater bar lengths.

For the case of bar-angle zero degrees (a line oriented orthogonally to its direction of passage, as in classical measurements of OP) no OP difference is seen until, as bar speed approaches wave speed, a 90° change in apparent OP takes place at a single increment in speed. This corresponds to transition to a “motion streak,” as object movement blurs resolution in the direction of motion. Increasing OP difference with bar speed at other bar angles is a more gradual development of the same effect—that is, mixing of responses to object speed and to object orientation. The illuminated field of supra-threshold excitation generated is not that expected to accompany a Gaussian-shaped tuning curve, but is roughly bimodal at medium speeds—e.g., bar-speed/wave-speed = 0.5. The form of the field of excitation is a combination of the classical preferred OP and the orthogonal orientation, expected as a consequence of Doppler shifts in the laterally-transmitted cortical signals generated by the moving visual input.

Variation of the window over which the apparent OP was estimated did not affect the qualitative results so long as averaging was conducted over a window beginning after the center point of the macrocolumn was crossed by the red bar. Variation of the estimate of wave speed was also without effect, so long as results were expressed in terms of bar speed/wave speed.

For comparative purposes similar simulations were performed in which contextual (green bar) responses were constrained to occur only with a limited angular response within a macrocolumn. That is, a restricted response to the line, according only to its orientation was imposed, in analogy to conventional models of OP, but with conduction delays of “like to like” fibers included. Then, systematic variation of OP with bar velocity did not occur.

These results match the findings of Basole et al. (2003) with respect to variation of OP peak responses as a function of line velocity and length. They do not reproduce the form of the experimentally observed Gaussian tuning curves, but as argued in the prior section, subsequent post-natal Hebbian learning progressively over-writing the Mobius configuration, and strengthening the peak response to the optimal visual signal, would concurrently strengthen responses to signals which are close to the optimum, resulting in Gaussian tuning curves in the more mature animals studied by Basole et al. (2003) and Issa et al. (2008).

### Inter-areal interactions of V1 and higher visual areas

The principle underlying the development of connections between macrocolumns and the superficial patch system may be generalized to the emergence of inter-areal connections. To recap, taking V1 as an example we have argued above that, because co-variance of activity declines with metric distance at both the scale of the patchy connections and within a macrocolumn, a homeotypic mapping between scales can emerge. This requires that relative distances on the maps at each scale must be in the ratio of correlation lengths of synchronous oscillation at the two scales, and adjacent maps must themselves have a correlated structure over a distance approximate to the correlation length of the patch system. It then follows that superposition of adjacent local maps, with appropriate rotation and correction to a common chirality, would result in a further map with co-variance of activity declining with metric distance, over the correlation length of the patch system.

Inter-areal connections, made by cortico-cortical axonal projections, could permit maps of this type to arise during ante-natal development, with the composite map at the higher cortical-area level itself folded into the Möbius configuration. The selection of saturated connections, projecting between areas with normalization of rotations and chiralities, would be possible by selection from the larger set of possible connections made by branches of cortico-cortical axons, diverging from their cells of origin to their cells of termination, overlapping as they terminate, and generally reciprocal between areas (Braitenberg and Schüz, 1991; Boucsein et al., 2011). Thus, antenatally, sets of macrocolumns at both the lower, V1, level and higher levels, could resonate with, and form preferential connections with, superimposed and overlapping groups at the other level, in accord with the developmental selection requirement to maximize joint synchrony. With the occurrence of eye-opening, Hebbian learning would then begin to overwrite the equilibrium resonance configuration between areas, in analogy to the process at intra-areal level—with the added property of associating concurrent patterns of activity in the V1 macrocolumns.

**Figure 8:**
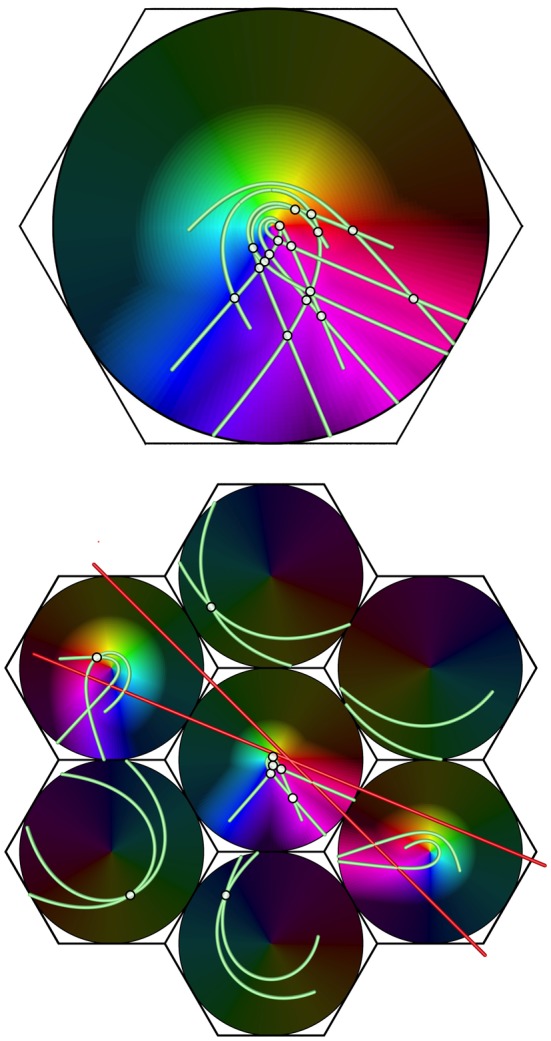
**Projection of a complex object (a double bar) from V1 (bottom) to a large macrocolumn in, e.g., V2 (top).** Brightened dots show the intersections of lines, producing more frequent enhanced responses to stimulus angles in V2 than V1.

Illustrating this effect, Figure 8 shows, at the bottom, a system of seven macrocolumns at V1 level, driven via the direct visual pathway by a pair of intersecting lines in the visual field.

The top part of the figure shows a projection of activity in conjointly activated macocolumns in V1, to a higher visual area, in which responses in the seven macrocolumns in V1 have been superimposed, with disparities in their orientation and chirality eliminated. Summations of points stimulated by both lines, shown by highlighted white points, occur frequently in the forward projection—much more so than at the level of V1 itself. These indicate response to angles of intersection of the lines at the lower level, and, commonly at the higher level, summating responses to time-lagged correlations between disparate positions of the moving visual stimulus. This effect is consistent with the preferential responses to angular and complex stimuli, characteristic of higher cortical levels (Merigan and Maunsell, 1993).

Conversely, since connections between higher and lower levels are generally reciprocal, a possible mechanism permitting control of attention (Rao and Ballard, 1997; Kveraga et al., 2007; Swindale, 2008; Naci et al., 2012) is suggested, since the backward flow would continuously modify the forward flow of sensory information.

## Conclusion

The model of cortical development we have outlined above is efficient from both energetic and information-processing perspectives, and has considerable anatomical and physiological explanatory power. It leads to an explanation of the spatial organization of signal flow in the cortex that differs from any other model. The proposed antenatal self-organization of cortical synapses leads to the creation of a *tabula rasa* on which homeomorphic maps, in a form disguised by the Möbius-strip-like folding of connections, occur in lateral connections at the millimetric scale, embedding the statistics of spatial organization of the sensory world to first approximation, before any detailed sensory inputs are received. The assumptions and findings of the model overlap with, and although not necessarily contradictory to, are not identical to, those of other models (Erwin and Miller, 1998; Wolf and Geisel, 1998; Kang et al., 2003; Oster and Bressloff, 2006). Distinguishing features include the explanation of the relationship of superficial patch connections to macrocolumn centers, and their hexagonal rotational symmetry, and crucially, the findings of Basole et al. (2003), which cannot be explained by any model dependent on “like-to-like” connections between feature-specific neurons. Nor can any model with otherwise similar assumptions about the self-organizing effect of synchrony be formulated without introducing a Möbius configuration to the connections, since an equivalent model utilizing only Euclidian conformations would represent a given OP twice, rather than once, around a singularity.

In review, the assumptions and conclusions reached, were as follows. By assuming that cells surviving apoptosis are selected by competition for metabolic substrates, and that synchronous oscillation mediates the uptake of metabolic substrates, we showed the outcome was a neural system with ultra-small world axonal configuration. Further assuming the small world connections were necessarily constructed from neuron populations characterized by respective axonal length, we showed that long range patchy connections and regular macro-column-like areas with central sparing of patchy connections emerge, with some degree of hexagonal rotational symmetry, with species variation in orderliness according to cortical size, and were able to show that this result was consistent with anatomical observations of limited interspecies variation of singularity density. A crucial further assumption made, was that metabolic competition between synapses from the same neuron leads to particular configuration of synaptic current flows at equilibrium, in which active connection networks within each macrocolumn are arranged in a Möbius-strip-like conformation. Then, with the introduction of visual inputs, signals conveyed by contextual fibers transfer a visual image from the global map to each local map, determining the pattern of neuron firing induced by activation of the cRF, and synaptic consolidation on Hebbian principles begins—thus storing information based on visual experience—explaining how response maps for OP, SF, and TF become organized in accord with the spatio-temporal filter model (Baker and Issa, 2005; Issa et al., 2008), and how “like to like” anatomical connections emerge, as well as providing conditions for dimension-reduction description of response features. The model is also compatible with explanation of ocular columns and direction preference fractures, as proposed in our earlier work (Wright et al., 2006).

The resulting synaptic storage of learned information in local topological maps of Möbius configuration offers a further compression of format, adding to the efficiency of the “small world” arrangement, by minimizing the distance which need be spanned by connections between positions on the local map representing positions widely separated on the global map. The development of cross-links also offers large potential information storage, since the regular spatial organization of links in the Möbius configuration implies the synaptic connections have low joint entropy in their ante-natal state. With visual experience, and the storage of image information in cross-links, joint entropy could, in principle, increase to a limit where all synaptic states are independent, and equally distributed about some mean connection strength, as implied by Montague's (1996) resource consumption principle. In effect, before eye-opening, the cortex has “learnt” the underlying statistical structure of visual space—that of cross-correlation declining with metric distance—and subsequently stores information about departures from this “first component” of structure in the visual world.

The antenatal development of response maps (Wiesel and Hubel, 1974; Blakemore and Van Sluyters, 1975; Sherk and Stryker, 1976) presents no paradox in this model, since emergence of organized response properties within the Möbius configuration does not depend upon structured visual stimuli. EEG activity progressively matures toward alternating alert and sleeping states in the later antenatal period (Marks et al., 1995; Mirmiran, 1995) providing the widespread co-ordination of pre-synaptic activity required for initial synaptic self-organization. Conversely, overwriting by learning in the immediate and later post-natal periods explains why representation in adult response maps of stimuli to which the subject as has not been exposed would not be present—as also seen experimentally (e.g., Blakemore and Van Sluyters, 1975).

No direct evidence yet exists of Möbius-like patterns of connections in cortex, yet this is scarcely surprising if transient dynamic couplings, present only in the equilibrium state, are overwritten by post-natal learning-related changes. However, relaxation toward the equilibrium condition is still to be expected in the mature state, so it is important whether or not some anatomical substrate exists in which the dynamic state of synapses may be capable of transient assembly into Möbius patterns. Markram and colleagues (Perin et al., 2011) found that pyramidal neuron networks cluster into multiple groups of a few dozen neurons each, with the neurons composing each group typically more than 100 μm apart, allowing for multiple groups to be interlaced in the same space. Connections within groups were largely reciprocal, and those between groups relatively sparse. Transient interlinkages between such interwoven linked groups could form Möbius-like networks. The temporal plasticity of synaptic connections near singularities (Dragoi et al., 2001) is also consistent with this interpretation. As well as plasticity of responses near OP singularities, Dragoi and colleagues found lack of plasticity in linear zones—the areas of strong patchy connection termination. This is to be expected if the patch system is composed of well consolidated connections suitable for consistent transmission with delay from fixed points in V1, while of the other hand, more complex, continually modified, information processing goes on in the areas around singularities. Consequently, an anatomical test of the model may be possible, in regard to the terminations of patchy connections in the periphery of the patch-free areas about singularities. As indicated in Figure 3, two populations of synaptic connections should be demonstrable in principle, by double injection/staining methods, near the singularity/patch edge. If some Hebbian consolidation occurs both antenatally and postnatally, then, in principle it should be possible to observe Möbius-like connections within macrocolumns antenatally, and the overwriting of these connections during post-natal learning.

If later testing supports this model, current conceptions of cortical information processing will require modification. Synchronous oscillation has been regarded as a mechanism for feature-binding—requiring that groups of cells in synchrony stand out in some way against a non-synchronized background. Instead, this model emphasizes synchrony as the organizer of a matrix of connections within which each macrocolumn gathers information from its surround, and organizes these connections systematically according to spatial position and time-lag, as functions of distance from each singularity. The topology of signal organization is markedly different to that of the association of “feature” neurons embedded in neural connections that are deployed on a Euclidean plane, as it implies that sensory images are not broken up into “features” which are subsequently associated in an abstract feature space, but retain, in modified form, an organization representing sensory space and time. Upon this more complicated matrix of connections, moment-by-moment states of autonomous local firing could interact with each other via traveling waves, generating internal images adding to those arising from sensation—all selectively strengthening preferred pathways by Hebbian learning, under the supervision of motivational systems. This gives a modified basis to Sherrington's “enchanted loom” (Sherrington, 1906, 1940), and a stage for the kind of neuro-dynamic events progressively observed and envisaged by Freeman for many years (Freeman, 1975; Freeman and Quiroga, 2013).

Hierarchical interaction of V1 with higher visual areas, by superposition of spatio-temporal images transmitted over convergent and divergent pathways might proceed to higher levels of abstraction, at higher cortical levels, and feedback interactions of ascending and descending signals in such a system might permit very complex image manipulation. Analogous processes may apply to other modalities throughout the cortex in general, since all sensory input systems are analogous to the visual system, in as much as they encode the sensory world by imposing a topological order to inputs as they arrive at the sensory cortices. Again, the ubiquitous distribution of patchy connections throughout the cortex, and the basic modular similarity of the paleo- and neocortex throughout, supports the notion that a single schema of information flow may be characteristic of all. The principle of organization might even extend to the motor cortex, with the efferent pyramidal motor neurons simply reversing the role of neurons in the direct visual pathway.

### Conflict of interest statement

The authors declare that the research was conducted in the absence of any commercial or financial relationships that could be construed as a potential conflict of interest.

## Supplementary material

The Supplementary Material for this article can be found on-line at http://www.frontiersin.org/Computational_Neuroscience/10.3389/fncom.2013.00004/abstract
